# Molecular modeling piloted analysis for semicarbazone derivative of curcumin as a potent Abl-kinase inhibitor targeting colon cancer

**DOI:** 10.1007/s13205-021-03051-9

**Published:** 2021-11-22

**Authors:** Fiona C. Rodrigues, Gangadhar Hari, K. S. R. Pai, Akhil Suresh, Usha Y. Nayak, N. V. Anilkumar, Goutam Thakur

**Affiliations:** 1 Department of Biomedical Engineering, Manipal Institute of Technology, Manipal Academy of Higher Education, Manipal, 576 104 India; 2 Department of Pharmacology, Manipal College of Pharmaceutical Sciences, Manipal Academy of Higher Education, Manipal, 576 104 India; 3 Department of Pharmaceutics, Manipal College of Pharmaceutical Sciences, Manipal Academy of Higher Education, Manipal, 576 104 India; 4 Department of Chemistry, Manipal Institute of Technology, Manipal Academy of Higher Education, Manipal, 576 104 India

**Keywords:** Abl kinase, Colorectal cancer, Curcumin, Heterocyclic, Molecular docking, Molecular dynamics

## Abstract

The human Abl kinases comprise a family of proteins that are known to be key stimulus drivers in the signaling pathways modulating cell growth, cell survival, cell adhesion, and apoptosis. Recent collative studies have indicated the role of activation of Abl and Abl-related genes in solid tumors; further terming the Abl kinases as molecular switches which promote proliferation, tumorigenesis, and metastasis. The up-regulated Abl-kinase expression in colorectal cancer (CRC) and the role of Abl tyrosine kinase activity in the Matrigel invasion of CRC cells have cemented its significance in CRC advancement. Therefore, the requisite of identifying small molecules which serve as Abl selective inhibitors and designing anti-Abl therapies, particularly for CRC tumors, has driven this study. Curcumin has been touted as an effective inhibitor of cancer cells; however, it is limited by its physicochemical inadequacies. Hence, we have studied the behavior of heterocyclic derivatives of curcumin via computational tools such as pharmacophore-based virtual screening, molecular docking, free-energy binding, and ADME profiling. The most actively docked molecule, 3,5-bis(4-hydroxy-3-methylstyryl)-1H-pyrazole-1-carboxamide, was comparatively evaluated against Curcumin via molecular dynamics simulation using Desmond, Schrödinger. The study exhibited the improved stability of the derivative as compared to Curcumin in the tested protein pocket and displayed the interaction bonds with the contacted key amino acids. To further establish the claim, the derivatives were synthesized via the mechanism of cyclization of Curcumin and screened in vitro using SRB assay against human CRC cell line, HCT 116. The active derivative indicated an IC_50_ value of 5.85 µM, which was sevenfold lower as compared to Curcumin’s IC_50_ of 35.40 µM. Hence, the results base the potential role of the curcumin derivative in modulating Abl-kinase activity and in turn may have potential therapeutic value as a lead for CRC therapy.

**Supplementary Information:**

The online version contains supplementary material available at 10.1007/s13205-021-03051-9.

## Introduction

Cancer can be defined as genetic damage that leads to the faulty division of cells and mutation, and according to the Global Cancer Statistics of 2020, it is still one of the leading causes of mortality (Sung et al. 2021). The new challenge of COVID-19 has led to emphasize the importance of strong immunity and the minimization of other comorbidities. Studies correlating cancer and COVID-19 have suggested that patients with cancer have a threefold mortality vulnerability due to COVID-19 (Pathania et al. 2021) (Dai et al. 2020). To develop and target cancer therapy and chemotherapy, elaborate investigations have been carried out to identify the underlying molecular basis of cancer and one such driver in the cascade is the Abelson (Abl) tyrosine kinases family. Many studies have ubiquitously suggested that Abl tyrosine kinases play a regulatory role in controlling cell growth, survival, invasion, adhesion, and migration (Colicelli 2010) (J. Wang and Pendergast 2015).

Abl genes are present in all metazoans and the family of protein kinases comprise closely associated paralogs Abl1 and Abl2 (also called ARG) which are highly conserved. The domains are majorly divided into the N-terminal accounting for the SH3 and SH2 and kinase domains whereas the C-terminal is associated with the actin-binding domain (ABD). The presence of multiple distinct domains in Abl1 and Abl2 modulates their differential subcellular localization as well as the ability to form unique protein complexes which cause the diverse functional roles in multiple cell types (Colicelli 2010; Wang 2014). Major studies have attributed the cause of chronic myeloid leukemia to the fusion gene formed as a result of BCR/Abl chromosomal translocation (Bedi et al. 1994; Ben-Neriah et al. 1986). The activation of Abl kinases promotes invasion and cellular cascades in the epithelial–mesenchymal transition (EMT) which further plays a role in metastasis for solid tumors (Luttman et al. 2021). Another study indicates that the kinases are promoters of metastasis of lung cancer cells exhibiting an EGFR or KRAS mutation (Gu et al. 2016). The definitive role and molecular mechanism of Abl kinases in colorectal cancer (CRC) was clinically evaluated by Liu et al. through several sequencing and gene expression analyses. It was revealed that elevated expressions of the Abl1 gene and elevated mutations were observed in CRC tissues and cell lines, as well as its involvement in the regulation of apoptosis in CRC cells, was established (Liu et al. 2020). Another study revealed that Abl tyrosine kinase activity played a significant role in Matrigel's invasion of human CRC cells. The study also tempered with DAB1 expression, which is phosphorylated by Abl tyrosine kinase and in turn activates Abl which amounts to promotion of CRC cell invasion (Sonoshita et al. 2015). There are existing selective and nonselective Abl-kinase inhibitors in the market, which are Imatinib, Dasatinib, Nilotinib, Bosutinib, Ponatinib, Axitinib, and Vandetanib (Wang and Pendergast 2015). However, multiple factors such as off-drug target effects, and intrinsic drug resistance exhibited by the existing drugs drive the research for the identification of alternative Abl-kinase inhibitors (Jones and Thompson 2020). Furthermore, the modulation of Abl overexpression, amplification, and mutation is a causative factor in solid tumors, and hence, there is a pressing need to direct drug discovery efforts to develop small molecule inhibitors of Abl kinases for cancer therapy.

The past few decades have identified a shift in the trend and have focused on finding the answers to the complexities of cancer in traditional medicine and curcumin has proven to be an excellent candidate in this study. Curcumin has had a repute for being a pleiotropic mediator of multiple signaling pathways which are crucial to the progression of cancer (Bhatia et al. 2021). In a previous study, curcumin has indicated the ability to inhibit proliferation and induce apoptosis of wild-type leukemia cells (T315I Bcl-Abl mutation) at small concentrations (William et al. 2008). Several other studies have also displayed curcumin's ability to induce apoptosis by regulation of Akt-mTOR, Abl/STAT5 pathway, Abl/p53, and Abl/JNK pathways (Guo et al. 2015; Guo et al. 2018; Kamath et al. 2007; Golonko et al. 2019). However, physicochemical limitations such as curcumin's hydrophobicity, reduced bioavailability, instability, and immediate riddance from systemic circulation hinder curcumins efficacy. A widely adopted strategy to improve the effectiveness of curcumin is by incorporating modifications to its structural scaffold by identifying potential reactive sites and many studies have also investigated its implications in the improvement of anti-cancer activity (Vyas et al., 2013; Rodrigues et al. 2019). One promising reaction site is the diketo moiety which has been held responsible for curcumins recurring instability, rapid metabolism, and poor solubility, for which studies have indicated that replacing it with a heterocyclic ring cyclization has led to the stabilization of the structure (Liang et al. 2009; Liao et al. 2019; Reddy et al. 2013). In the domains of medicinal chemistry, multiple reports have elaborated the enhancement of curcumin's structural behavior and applicative aspect on replacement with a heterocyclic moiety (Rodrigues et al. 2021).

With the advancements in medical technology, the different approaches, strategies, and methodologies for drug design are being constantly revamped to solve the setbacks of traditional drug discovery which are majorly cost and time-based. Apart from in vitro and in vivo experimentation, computer-aided drug design has been expansively explored and its role in drug discovery and drug repurposing has been of paramount significance in the past few decades. Medicinal chemistry has found structure-based drug design (SBDD) as a promising approach to achieve specificity against a target, and it engages the concepts of flexibility of the receptor, conformational accountability, structure–activity relationships analysis, and pharmacophore-based virtual screening (Kalyaanamoorthy and Chen 2011). Multiple studies have resorted to drug repurposing for screening in drug discovery and computational methods of docking and Artificial Intelligence have proven to be of paramount significance, particularly in medicinal plant research (Singh and Bharadvaja 2021). Furthermore, molecular modeling attempts to mimic the biological system by virtual-screening methodologies and binding energy predictions, hence playing an essential role in the advancement of pharmaceutical therapeutics (Durrant and McCammon 2011). In this context, we attempt to closely study the rigid and flexible docking of the designed heterocyclic curcumin derivatives as ligands against the human Abl-kinase protein and carry out a comparative analysis with curcumin. Furthermore, the compounds which have indicated promising docking scores and free-energy binding calculations will be evaluated for their flexible binding capabilities to recognize the stability of the ligand and the compounds will also be synthesized and evaluated in vitro to understand their cytotoxic potential. To the best of our knowledge, this report is the first of its kind to scrutinize and identify Abl-kinase inhibitors using receptor–inhibitor complex for these compounds based on computational analysis.

## Materials and methods

The licensed commercial software-Maestro Molecular platform (Version 2021–3) by Schrödinger (Schrödinger, LLC, New York) was used to facilitate molecular modeling and docking on an HP computational set-up with a Linux Ubuntu 18.04.1 LTS operating system. Furthermore, Molecular dynamics simulation studies were implemented with the Intel® Xenon(R) Gold 6130 CPU @ 2.10 GHz × 64 processors, Quadro P620/PCle/SSE2 graphics card, and 134.8 GB RAM.

### Protein preparation and receptor grid generation

The crystal structure of Human Abl kinase in complex with nilotinib (PDB ID – 3CS9) has a resolution of 2.21 Å (Weisberg et al. 2005) and was retrieved from the RCSB protein data bank (https://www.rcsb.org) (Berman 2000). Preparation of the protein was implemented by the ‘Protein Preparation Wizard’ in the Schrödinger suite during which the protein was refined, modified, and minimized (Madhavi Sastry et al. 2013). The process identifies and fills missing side chains, hydrogen atoms, and residues via the Prime tool, as well as rectifies water molecules, heavy atoms, cofactors, and metal ions. To generate the most stable energy state, energy minimization for the protein was performed using the OPLS3e force field (Banks et al. 2005)(Harder et al. 2016). For grid generation, a cubicle grid was set-up around the active site of the protein to keep all the functional amino acid residues bound.

### Ligand preparation

The designed curcumin analogues were constructed via the 2DSketcher tool. For the ligand optimization, the LigPrep tool was employed using the OPLS3e force field to generate the lowest energy 3D structures. All ligands were pre-processed, tautomers were generated and default parameters were selected (Chen and Foloppe 2010)(LigPrep 2015).

### Molecular docking

To generate the molecular docking data, the GLIDE operational ligand docking tool of Maestro was used (Friesner et al. 2004). As the designed analogues and generated tautomers were small in number, they were screened using the extra precision (XP) method as it uses descriptor and explicit water technology. The XP method also clears out false positives and employs a protocol with a refined growth strategy (Kumar et al. 2020). The best-docked pose, XP score, and GLIDE Score were recorded for each ligand (Elokely and Doerksen 2013).

### Free ligand-binding energy calculation (MM-GBSA)

The Prime module of Maestro was operated to investigate the absolute ligand-binding affinities of the designed heterocyclic curcumin analogues using MM-GBSA (molecular mechanics energies generalized Born and surface area continuum Solvation) method (Genheden and Ryde 2015). The MM-GBSA assay was performed via the Pose viewer file of GLIDE XP mode by the VSGB solvation model and OPLS3e force field to refine the binding energy calculations.

### ADME profile

The ADME (Absorption, Distribution, Metabolism, and Excretion) profile of the selected compounds was carried out via the QikProp module (QikProp). The compounds were comparatively analyzed for certain parameters based on the pre-determined cut-off values to predict the druggable property. Some of the parameters studied were the Lipinski rule of five and various descriptors like QPlogHERG, QPPCaco, QPlogBB, % human oral absorption among others (Leeson 2012). Apart from ADME characteristics, the listed compounds were also comparatively evaluated for other descriptors which evaluated the variations in the solvent-accessible surface areas (Dasari et al. 2017). These values are indicative of the transferring free energy on movement from a polar medium to a nonpolar medium (Lee and Richards 1971). The other evaluated parameters were FISA, FOSA, PISA, and PSA.

### Molecular dynamics

After understanding the rigid docking of the ligand–protein complex, its flexible docking was studied via molecular dynamics (MD) to mimic the biological system. MD simulations were implemented via the Desmond tool of Schrödinger Drug Design Suite. Based on the docking score and free binding energy, two ligands were carried forward for MD simulation for 50 ns to study their stability. The three steps performed for MD simulation were building the system, minimization, and the MD simulation itself. The docked ligand–protein complex was selected, and the system was modeled by a predefined solvent system—SPC under the orthorhombic boundary conditions. Any negative charges on the model were neutralized with sodium ions and the model was subjected to energy minimization until a gradient threshold of 25 kcal/mol/Å was achieved at a temperature of 300 K and 1 bar pressure via NPT ensemble class. On conduction of the MD simulation, the trajectory was recorded and the stability of the complex was evaluated by the Protein and Ligand RMSD (Root-Mean-Square Deviation) fluctuations, Protein–Ligand interactions, and contacts with various amino acids using the Simulation Event Analysis tool of Desmond (Bowers et al. 2006).

### Synthesis and characterization of the designed compounds

The synthesis of the designed compounds was enabled via a simple one-pot condensation method in which Curcumin was treated with suitable substituted primary hydrazines and pyrimidine to obtain the studied pyrazole and isoxazole derivatives. The compounds were characterized for their structural integrity by FTIR, ^1^H NMR, ^13^C NMR, DSC, and LC–MS. The elaborated synthesis and characterization of these compounds have been reported in our previous study (Rodrigues et al. 2021).

### Cell viability assay

#### Cell culture

The human colorectal cancer cell line, HCT 116, was procured from the National Centre of Cell Science, Pune, India. The cells were cultured in DMEM (Dulbecco's Modified Eagle's Medium) supplemented with 10% (v/v) FBS (Fetal Bovine Serum), and with 2% Antibiotic–Antimycotic solution. The cells (5 × 10^3^ cells/well) were seeded in sterile 96 well plates and were allowed to adhere to the plate for 24 h at 37 °C in a humidified atmosphere (90%) containing 5% CO_2_.

#### SRB assay

The IC_50_ values of the synthesized curcumin analogues were determined by Sulforhodamine B (SRB) assay. The assay was performed in accordance to formerly described protocols with slight modifications (Houghton et al. 2007) (Vichai and Kirtikara 2006) (Rodrigues et al. 2021). The incubated HCT 116 cells were treated with the test compounds at concentrations ranging from 500 to 7.81 µM for 48 h. Dimethyl sulfoxide (DMSO) was assigned as the vehicle control and Doxorubicin was the positive control as the model drug. To record the absorbance, a scanning multiplate reader (ELx800, BioTek Instruments Inc., Winooski, VT, USA) was used at 540 nm, and the percentage cell viability was calculated using an excel sheet, and IC_50_ values were determined using Graph Pad Prism.

### Statistical analysis

Statistical analysis of data was performed using a one-way t test to determine p values and the results indicating *p* < 0.05 were estimated to be significant. Origin 6.0 and Graph Pad Prism V.8 software was used for analysis.

## Results

### Ligand docking

Rigid docking was carried out for curcumin, its generated tautomers, and the designed derivatives. The design of the curcumin analogues was targeted to alter the diketo site as it has been attributed for the instability of the molecule and to stabilize the scaffold a ring cyclization in the form of isoxazole and pyrazoles has been investigated. In total, the seven ligands were screened with the extensive XP docking mode of the GLIDE panel. The XP mode was the selected mode of docking as the studied data set was limited, and the XP mode delivers an accurate estimation of the good pose of drugs and corresponding score along with lower chances of a false positive result as compared to the other modes. All the ligands screened indicated docking scores ranging between  – 12.064, which was the highest to  – 6.539 which was the lowest recorded docking score belonging to curcumin-N-amido pyrazole and curcumin-N-phenyl pyrazole, respectively. The remaining compounds, which were Curcumin, its tautomers, and the isoxazole, pyrazole counterparts indicated similar docking scores ranging between  – 10.889 and  – 10.042. Other Docking scores and GLIDE scores were in congruence with the XP docking scores indicating very little difference in the data sets (Table 1).

**Table 1:** Docking score and prime MM-GBSA score of titular compounds

S. no.	Ligand	Docking Score	XP Score	GLIDE Score	MM-GBSA ΔG bind (Kcal/mol)
1	Curcumin	– 9.979	– 10.889	– 10.889	– 57.33
2	Curcumin Tautomer 1	– 9.656	– 10.323	– 10.323	– 56.50
3	Curcumin Tautomer 2	– 8.624	– 10.042	– 10.042	– 72.61
4	Curcumin Isoxazole	– 10.864	– 10.864	– 10.864	– 63.53
5	Curcumin Pyrazole	– 10.421	– 10.421	– 10.421	– 65.58
6	Curcumin Semicarbazide	– 12.064	– 12.064	– 12.064	– 70.27
7	Curcumin-2,4 DNPH	– 6.539	– 6.539	– 6.539	– 47.05

On analysis of the 2D ligand-interaction diagrams from Table 2 and dG bind scores from Table 1, it was elucidated that five of the seven tested ligands indicated H-bonding interaction with MET 318. The highest XP dock score ( – 12.064) was indicated by the Curcumin Semicarbazide which had another H-bond interaction with MET 318 as well as ILE 313. The ligand also depicted a pi–pi interaction with PHE 382 residue. The ligand has shown a promising dG bind score of  – 70.27 kcal/mol. The highest dG bind score was indicated by a tautomer of curcumin with a score of  – 72.61 kcal/mol; however, it has indicated a relatively poor XP docking score and has also indicated only a single pi–pi stacking interaction with TYR 253 residue. The next highest XP docking score was indicated by curcumin ( – 10.889), with a dG bind score of  – 57.33 kcal/mol and one H-bond interaction with MET 318. Curcumin isoxazole and curcumin pyrazole have indicated similar XP docking scores of  – 10.864 and  – 10.424, respectively, and identical interactions of H bond with MET 318 and pi–pi stacking with TYR 253, PHE 382 residues. These compounds have also indicated a similar dG score of 63.53 kcal/mol and  – 65.58 kcal/mol. The compound indicating the least docking score and dG bind score of  – 6.539 and  – 47.05 kcal/mol was Curcumin-2,4 DNPH. Ironically, this compound has indicated multiple uncommon interactions which are H-bond interactions with GLU 239, ARG 396 residues, pi–cation interactions with LYS 231, ARG 396 residues, and salt bridges with LYS 285, GLU 282 residues (Table 2). Based on the comparative analysis of the docking scores, dG binding scores, and protein–ligand interactions, curcumin, and its analogue, curcumin semicarbazide were chosen for further molecular dynamics and ADME profile studies.

**Table 2:** 2D interaction diagrams of titular compounds with a summary of all non-bounding interactions

S. no.	Ligand	IUPAC Name	2D ligand-interaction diagram	Interaction
1	Curcumin	1,7-Bis-(4-hydroxy-3-methoxy-phenyl)-hepta-1,6-diene-3,5-dione		H bond—MET 318
2	Curcumin Tautomer 1	5-Hydroxy-1,7-bis-(4-hydroxy-3-methoxy-phenyl)-hepta-1,4,6-trien-3-one		H bond—MET 318, GLU 286 Pi–pi stacking—TYR 253
3	Curcumin Tautomer 2	5-Hydroxy-1,7-bis-(4-hydroxy-3-methoxy-phenyl)-hepta-1,4,6-trien-3-one		Pi–pi stacking—TYR 253
4	Curcumin Isoxazole	4-[(1E)-2-[3-[(1E)-2-(4-hydroxy-3-methoxyphenyl)ethenyl]-5-isoxazolyl]ethenyl]-2-methoxyphenol		H bond—MET 318 Pi–pi stacking—TYR 253, PHE 382
5	Curcumin Pyrazole	4,4'-[(1E)-1H-Pyrazole-3,5-diyldi-2,1-ethenediyl]bis[2-methoxyphenol]		H bond—MET 318 Pi–pi stacking—TYR 253, PHE 382
6	Curcumin Semicarbazide	3,5-Bis[(1E)-2-(4-hydroxy-3-methoxyphenyl)ethenyl]-1H-pyrazole-1-carboxamide		H bond—MET 318, ILE 313 Pi–pi stacking—PHE 382
7	Curcumin-2,4 DNPH	4,4'-[[1-(2,4-Dinitrophenyl)-1H-pyrazole-3,5-diyl]di-(1E)-2,1-ethenediyl]bis[2-methoxyphenol]		H bond—GLU 239, ARG 396 Pi–cation—LYS 231, ARG 396 Salt bridges – LYS 285, GLU 282

### ADME

Before proceeding for the molecular dynamics simulation study, the two selected ligands indicating the highest docking score and in turn being promising inhibitors were tested for their drug-like properties. Analyzing ADME properties of the selected ligands enhances lead optimization and reduces the exaggerated attrition rate in the drug discovery process. The physicochemical parameters were analyzed via the QikProp tool of Maestro and the results of the drug-likeness are compiled in Table 3. All the descriptors analyzed were within the acceptable range, have indicated no violations of Lipinski’s rule of five, and have indicated acceptable oral absorption. Furthermore, the star parameter has indicated a zero score for both ligands, hence, implying that the two ligands have drug-like properties similar to 95% of known drugs.

**Table 3:** Drug likeness analysis of the two selected ligands

Ligand	Star	MW	HB_d_	HB_a_	QPlog Po/w	QPlog HERG	%Human Oral absorption	Rule of five
Curcumin	0	368.385	2	7	2.812	– 6.312	82.343	0
Curcumin Semicarbazide	0	407.425	4	6	2.499	– 5.356	70.791	0
Recommended values	0–5	130–725	0–6	2–20	– 2 to 6.5	Concern below -5	> 80% is high < 25% is poor	Max. 4

*MW molecular weight, *HBd* predicted number of hydrogen-bond donor, *HBa* predicted number of hydrogen-bond acceptor, *QPlogPo/w* predicted octanol/water partition coefficient, *QPlogHERG* predicted IC_50_ value for the blockage of HERG Kþ channels, Rule of five, number of violations of Lipinski’s rule of five

Furthermore, the solvent-accessible surface area of these ligands and their further hydrophobic and hydrophilic complement parameters are elaborated in Table 4. Apart from that, the Van der Waals surface area of polar nitrogen and oxygen atoms has also been mentioned and all these values lie within the range of recommended values. Based on the data collated in Tables 3 and 4, it can be concluded that both these ligands have drug-like properties as all the descriptors screened for are within the permissible range.

**Table 4:** SASA, FISA, FOSA, PISA, and PSA calculations of ligand molecules

Ligand	SASA	FISA	FOSA	PISA	PSA
Curcumin	707.07	191.988	261.227	253.855	112.723
Curcumin Semicarbazide	746.148	224.722	214.55	306.875	127.05
Recommended values	300.0–1000.0	7.0–330.0	0.0–750.0	0.0–450.0	7–200

*SASA total solvent-accessible surface area (SASA) in square angstroms using a probe with a 1.4 Å radius, FISA, hydrophilic component of the SASA; FOSA, hydrophobic component of the SASA; PISA, π (carbon and attached hydrogen) component of the SASA; PSA, Van der Waals surface area of polar nitrogen and oxygen atoms and carbonyl carbon atoms

### Molecular dynamics

Within the bodily functionalities, the protein does not remain motionless to accommodate the incoming ligand to its receptor. There are constant random conformational rearrangements that require the testing of flexible docking of the protein and ligand. Molecular dynamics simulations facilitate flexible docking by applying Newtonian physics to simulate movements at an atomic scale. These simulations are represented in dynamic solvent environments, thereby mimicking an environment similar to the physiological system. In this study, MD simulation was carried out for the ligand indicating the best docking score, and ΔG bind, Curcumin semicarbazide was comparatively analyzed against Curcumin. This MD simulation study was carried out to get insights into the ligands' binding stability, and non-bonding interactions with crucial amino acids within the pocket of the protein.

**Fig. 1:**
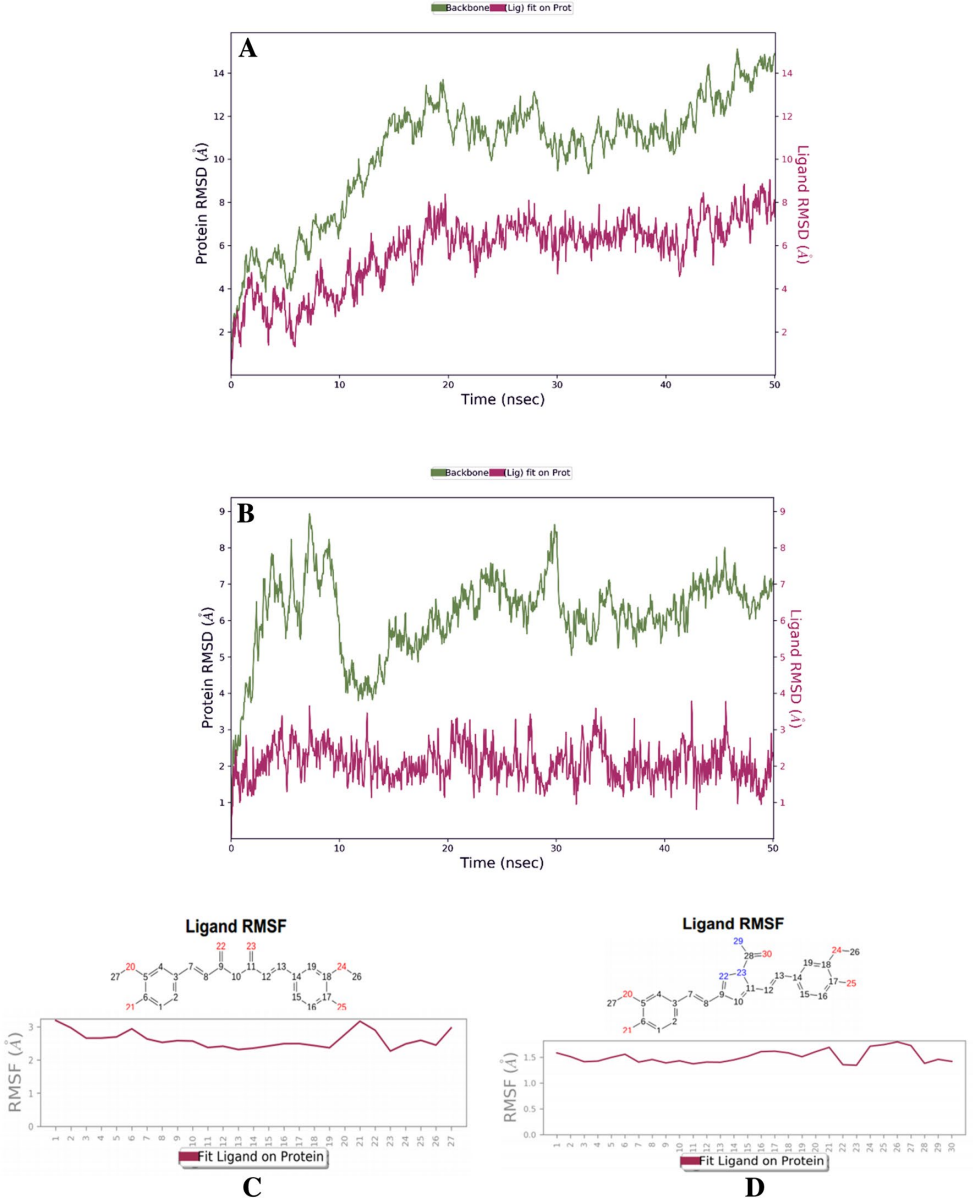
**A** Protein–ligand root-mean-square deviation (RMSD) plot of Curcumin bound to the inhibitory site of Abl-kinase protein. **B** Protein–ligand RMSD plot of Curcumin Semicarbazide bound to the inhibitory site of Abl-kinase protein. **C** Ligand RMSF of Curcumin **D** Ligand RMSF of Curcumin Semicarbazide

The simulation was carried for 50 ns using the Desmond tool and was comparatively analyzed between Curcumin and Curcumin semicarbazide. These ligands were chosen to be studied based on the molecular docking scores, binding energy, and as well as the absence of any violation of the recommended values of ADME. Several parameters such as RMSD of the protein and ligand, RMSF of the ligands and proteins, their respective torsion profiles, and the histogram indicating the interaction between the ligand and amino acids have been plotted and evaluated (Supplementary Information & Fig. 1). It was observed that when Curcumin is in the system, RMSD values of protein and ligand were 14.4 Å and 8.5 Å, respectively. A stable RMSD of 6.3 Å for the complex could be observed for the duration between 3 and 8 ns, after which a drift and fluctuation could be observed from 9 to 50 ns (Fig. 1A). In the case of Semicarbazide curcumin, RMSD values of protein and ligand were significantly lower, i.e., 7.8 Å and 2.6 Å, respectively, and the ligand RMSD was found to be within the acceptable range (1–3 Å). An initial drift was observed till 10 ns, after which a certain stabilization could be observed post-10 ns up till 50 ns (Fig. 1B). It was also observed that the protein was not under too much stress for both the tested ligands. Concerning the derivative complex as compared to the Curcumin complex, the difference in fluctuations could be observed for both ligands with a better complex stabilization in the derivative Curcumin Semicarbazide. Ligand RMSF is indicative of the ligands fluctuation atom-by-atom. The average Ligand RMSF for Curcumin was 3 Å, whereas the average Ligand RMSF for Curcumin Semicarbazide was 1.5 Å (Fig. 1C and D); this could be an indication of the less fluctuation recorded for the Curcumin-Semicarbazide complex when compared to the Curcumin complex. The results are indicative of a more stable ligand–protein complex, confirming that Curcumin Semicarbazide bound to the protein, performed as a better competitive inhibitor than Curcumin.

**Fig. 2:**
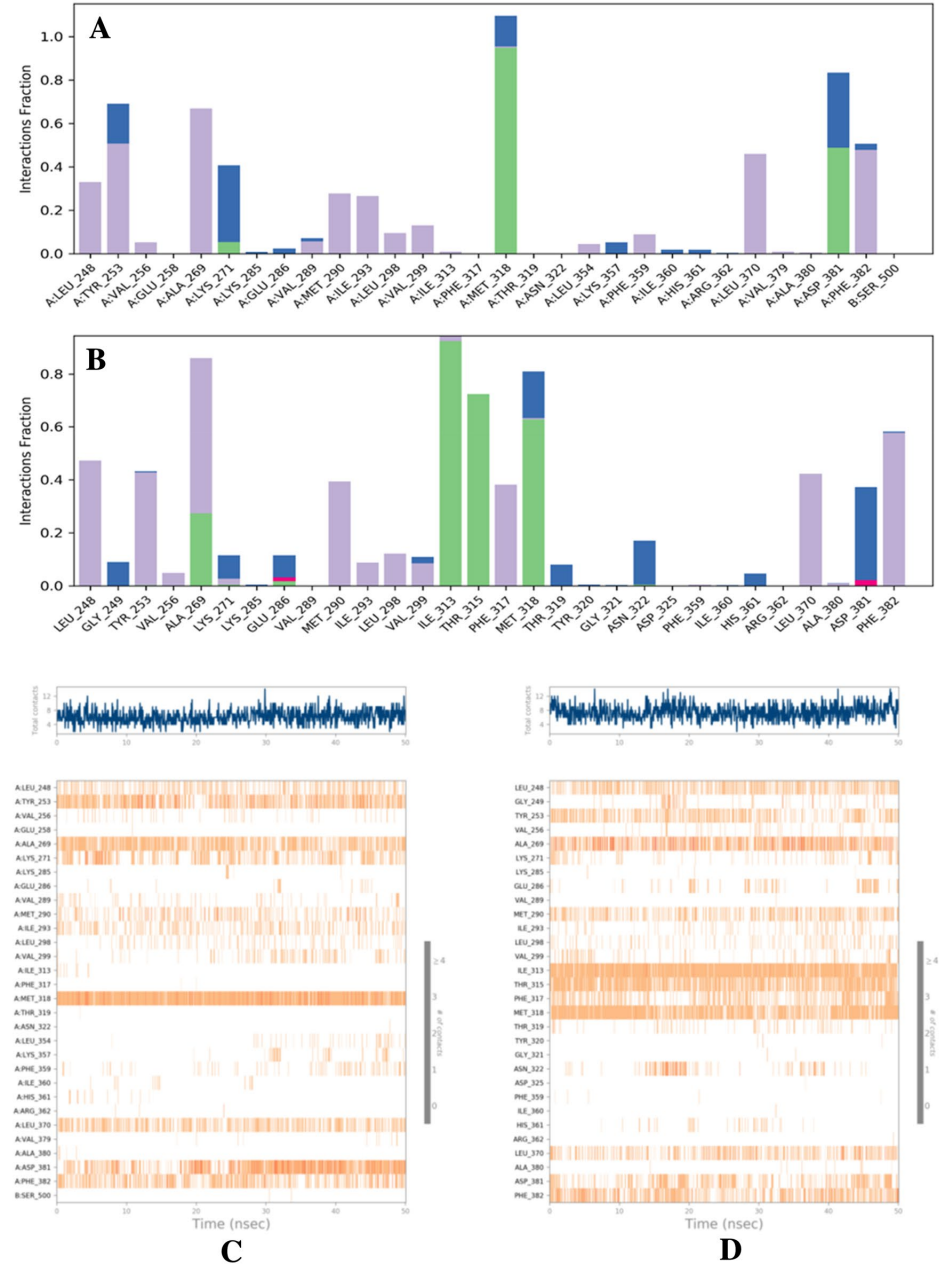
**A** Histogram of the protein–ligand complex of Curcumin and Abl-kinase protein. **B** Histogram of the protein–ligand complex of Curcumin semicarbazide and Abl-kinase protein. **C** Protein–ligand contact timeline plot of Curcumin bound to Abl-kinase protein. **D** Protein–ligand contact timeline plot of Curcumin Semicarbazide bound to Abl-kinase protein

To assess the interaction of the ligands with the protein, each contact with the amino acid was evaluated via the protein–ligand contact histogram and contact timeline plot (Fig. 2). It was noted that over the normalized course of the trajectory, in the Curcumin complex, only seven amino acids, i.e., TYR 253, ALA 269, LYS 271, MET 318, LEU 370, ASP 381, and PHE 382, had 40% of the simulation time with the specific interactions maintained (Fig. 2A & C). Whereas the Semicarbazide complex had 11 amino acids, i.e., LEU 248, TYR 253, ALA 269, MET 290, ILE 313, THR 315, PHE 317, MET 318, LEU 370, ASP 381, and PHE 382, had 40% of the simulation time with the specific interactions maintained. Interestingly key amino acids ALA 269, ILE 313, THR 315, MET 318, and PHE 382 had over 60% interactions and established strong contacts which are pivotal for stabilization. The derivative complex also indicated 2 ionic bond formations with GLU 286 and ASP 381 which was not observed in the Curcumin complex. This could be reflected in the contact timeline plot as well as a majority of these crucial bonds were indicated as hotspots (Fig. 2B and D).

### Synthesis of the designed compounds

The heterocyclic derivatives of curcumin were synthesized as per the previously reported study. The synthesis was carried out by attacking the central 1,3 β diketone reactive site of the curcumin scaffold to facilitate cyclization via condensation. The heterocyclic curcumin derivatives were further characterized for their structural integrity and purity by the routine characterization techniques (Rodrigues et al. 2021).

### SRB assay and cell viability

**Fig. 3:**
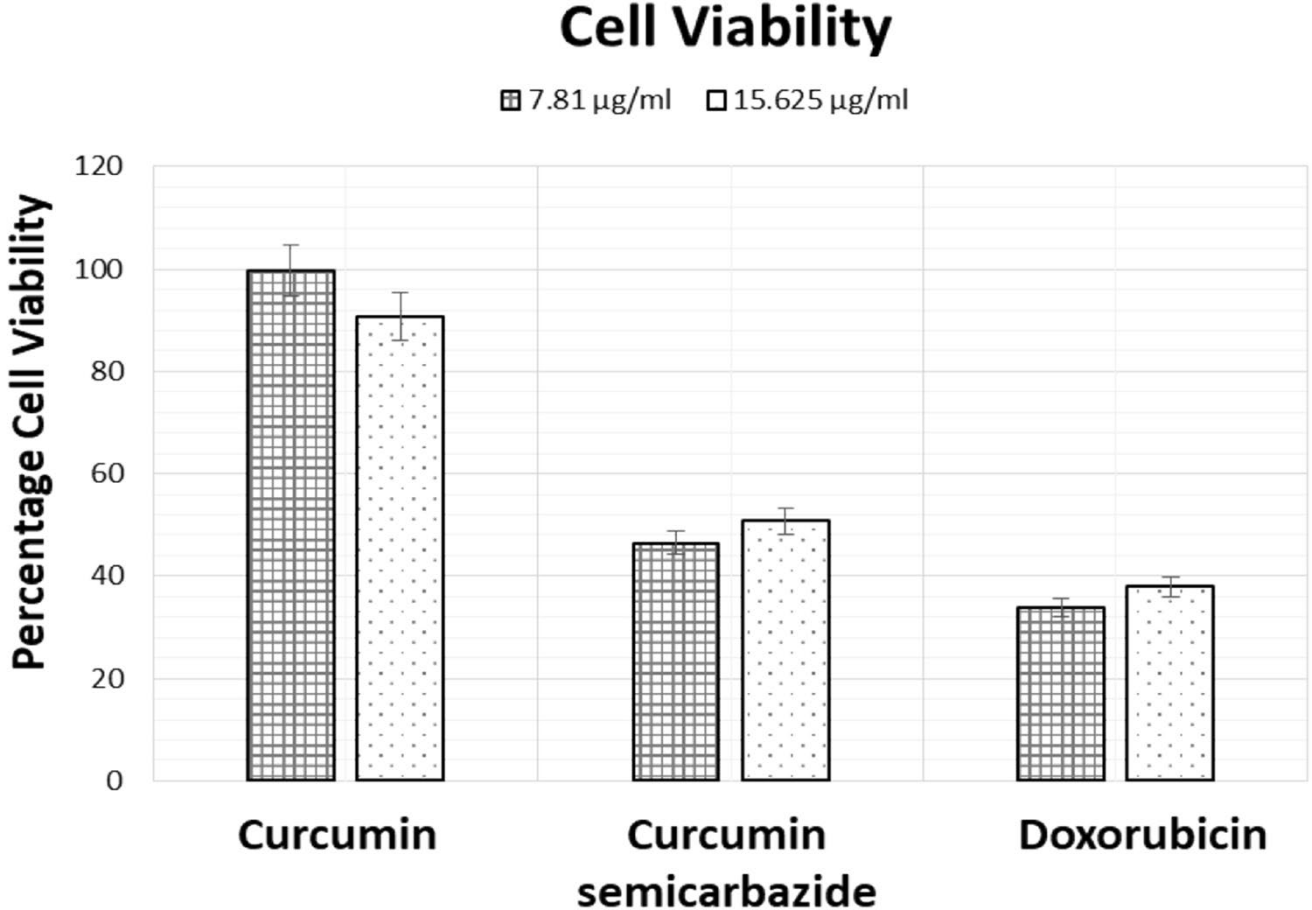
Effects of Curcumin, Curcumin-semicarbazide and Doxorubicin on cell viability. (A Significant statistical difference of *p* < 0.05 was found by performing *t*-test.)

The IC_50_ values of the synthesized compounds were calculated via SRB assay against HCT 116-human colon cancer cell line and Doxorubicin was taken as the standard control. Furthermore, cell viability for specific concentrations was comparatively analyzed for Curcumin, Curcumin semicarbazide, and Doxorubicin. The calculated IC_50_ value of Curcumin was recorded to be 35.40 µM and Doxorubicin indicated a value of 1.21 µM. Among the synthesized derivatives Curcumin isoxazole and curcumin-2,4 DNPH indicated poor IC50 values, even higher than curcumin. Whereas Curcumin pyrazole and Curcumin semicarbazide indicated IC_50_ values of 16.71 µM and 5.85 µM, respectively. Interestingly, curcumin semicarbazide indicated a sevenfold potency as compared to Curcumin for the in vitro screening (Table 5). The comparative analysis for the cell viability of Curcumin, Curcumin semicarbazide, and Doxorubicin was evaluated at 2 lower concentrations, i.e., 7.81 µg/ml and 15.625 µg/ml. Curcumin displayed very high cell viability for the studied concentration ranging above 90% and, hence, is not very effective at lower doses. Doxorubicin, on the other hand, displayed impressive cell viability of 34–38% at these concentrations. Curcumin semicarbazide indicated cell viability between 46 and 50% at the tested concentrations, which is highly effective when compared to Curcumin and satisfactorily adequate when compared to Doxorubicin (Fig. 3).

**Table 5:** IC_50_ values of synthesized compounds

S. no.	Compound name	IC_50_ (µM)
1	Curcumin	35.40
2	Curcumin-Isoxazole	48.53
3	Curcumin-Pyrazole	16.71
4	Curcumin-Semicarbazide	5.85
5	Curcumin-2,4 DNPH	> 80
6	Doxorubicin	1.21

## Discussion

It has been observed that there is a paradigm shift from modern medicine to traditional medicine in the quest for solutions to the complexities of diseases, particularly in diseases like cancer. Curcumin has been at the forefront of research in this domain and its pleiotropic behavior in modulating various cascades and molecular targets works to its advantage (Zhou et al. 2011). Its efficacy is limited by certain physicochemical parameters such as its solubility, bioavailability, and rapid elimination from the body; however, many attempts are being made to improve the effectiveness of curcumin (Nelson et al. 2017). Many studies have repeatedly implied that curcumin owes its instability to the diketo moiety and targeting this site for modification could lead to derivatives/analogues with more stability and better activity, respectively (Rajasekar Reddy et al. 2013) (Narlawar et al. 2008). Replacing the diketo group by introducing a heterocyclic ring scaffold to the skeletal structure of curcumin has led to improved stability and improved anti-proliferative activity (Rodrigues et al. 2021). Hence, to stabilize curcumin and enhance its pharmacodynamics profile, the designed and synthesized compounds are heterocyclic derivatives of curcumin and represent the main groups of most commonly tested compounds, i.e., isoxazole curcumin, pyrazole curcumin, N-amido pyrazole curcumin, and N-phenyl pyrazole curcumin. In this study, these titular compounds were evaluated to be Abl-kinase inhibitors as the Abl family of proteins are known to influence growth, survival, invasion, and angiogenesis during tumor initiation and progression (Lin and Arlinghaus 2008). Apart from its established role in chronic myeloid leukemia, studies have also suggested that amplification or overexpression of Abl1 and Abl2 is related to different carcinomas and related to solid tumors (Greuber et al. 2013). In a study carried out by Sonoshita et al., the cancer invasion-metastasis cascade was carefully evaluated for colorectal cancer via gene expression analysis. The study identified the signaling mechanism linking the Abl-kinase autophosphorylation and activation as a stimulant of colorectal cancer progression via the modulation of various receptors (Sonoshita et al. 2015). Furthermore, another study elucidated the role of Abl 1 in CRC gene mutation and found out that a high level of Abl1 expression was observed in CRC cells. A particular mutation of C1222C deletion in the Abl gene was found to be related to a CRC stage and the depletion of Abl1 was associated with proliferation inhibition in vitro when tested in two CRC cell lines, namely, HCT 116 and SW480. This elaborate study highlighted the significance of Abl1 as a potential target in CRC therapy by testing in vitro, in vivo as well as with tissue samples obtained from patients with CRC (Liu et al. 2020). The deliberations of these studies have led to the motivation and foundation of our work. In this study, we have empirically investigated the in silico profile of the designed curcumin analogues for their potency as Abl-kinase inhibitors via rigid as well as flexible docking profiles and have further screened the compounds for cytotoxicity in vitro. To the best of our knowledge, this has been the first extensive study of the listed compounds against the Abl-kinase protein in silico.

The influence of computational structure-based drug design has steered the process of drug discovery in a new direction owing to the development of precise computational architecture and accurate algorithms. The conformation, flexibility, and binding of the receptor–ligand complex are crucial to structure-based drug design and play a fundamental role in optimization for drug design (Meng et al. 2011). To analyze the designed ligands, the structures were subjected to docking, out of which the highest XP scores were recorded for semicarbazide curcumin and curcumin of  – 12.064 and  – 10.889 (Table 1), and hence, these were the chosen ligands were consequent analysis. Furthermore, the interactions that were observed for curcumin were MET 318 and for semicarbazide curcumin were hydrogen bonds with MET 318 and ILE 313 and pi–pi stacking with PHE 382 which is indicative of stable binding (Table 2). To further support the study, the binding energy depicted strong affinity and the studied ADME profile of the compounds has not indicated any violation of drug-likeness (Tables 3 and 4). To aid the claims, the two ligands were comparatively evaluated by their flexible docking via MD simulation and the results indicated that the curcumin-semicarbazide complex was more stable than the curcumin complex (Fig. 1). Both the compounds have indicated the formation of an H bond with MET 318 with the hydroxyl group of the curcumin and derivative scaffold which has been previously observed as a key interaction (Parcha et al. 2017). The additional hydrogen-bond interaction formed by the curcumin-semicarbazide complex was ILE 313 which has been previously reported to be favorable interaction for the complex (Zhou et al. 2016). The other hydrophobic, H bond, ionic, and water bridge interactions such as PHE 382, PHE 317, ASP 381, LEU 370, GLU 286, etc. have also been previously identified and established with the ligand and may be attributed to the stability of the protein–ligand complex (Faryna and Kalinichenko 2019) (Parcha et al. 2017) (Weisberg et al. 2005) (Kalinichenko et al. 2019) (Fig. 2).

The designed ligands were then synthesized pertaining to our previously reported study via a simple one-pot synthesis method exploiting the mechanism of condensation and were characterized for their structural integrity and purity by the routine characterization techniques (Rodrigues et al. 2021). The synthesis of these heterocyclic compounds has been of great interest to researchers as they exhibit a wide array of improved activities when compared to curcumin. The semicarbazide derivative of curcumin, in particular, has been previously synthesized and tested for multiple activities. Dutta and group initially identified the exceptional anti-oxidant potency of curcumin semicarbazone while conjugated with copper (Dutta et al. 2001). Furthermore, the group investigated Curcumin semicarbazones anti-proliferative and anti-oxidant activity; and identified that semicarbazones electron-withdrawing group as well as imine carbonyl and phenoxyl radical impart curcumin semicarbazone its anti-oxidant potential (Dutta et al. 2005). Furthermore, the compound has been tested for its anti-inflammatory, antinociceptive, cyclooxygenase-2 inhibition, carbonic-anhydrase inhibition, and anti-malarial (Ahmed et al. 2018a, b) (Ahmed et al. 2018a, b-2) (Balaji et al. 2019). To assess its cytotoxic potency and assert the claims put forth by the in silico analysis, the ligands were screened via SRB assay in human CRC cell line HCT 116 and semicarbazide curcumin indicated a sevenfold better activity than curcumin and also comparable dose-dependent cell viability at lower concentrations when compared to Doxorubicin (Table 5 and Fig. 3). These results are in congruence with previously reported results, such as the study conducted by Ahsan and team, in which semicarbazide curcumin and other analogues were tested for their binding interactions with EGFR tyrosine kinase and were further tested against multiple cell lines in vitro. The semicarbazide curcumin analogue resulted to be the most potent compound in this study and had indicated superior activity in over 47 cell lines inclusive of colon cancer cell lines, as well as a better activity than the standard drug—paclitaxel in 42 cell lines. The retaining of phenyl hydroxyl groups proved to be an important factor for EGFR-TK binding and molecular docking analysis revealed the strong hydrophobic interactions of the compound which was reflected in its in vitro anti-proliferative potency (Ahsan et al. 2015).

## Conclusion

In conclusion, the current study facilitated the identification of a potent heterocyclic curcumin analogue that could behave as a better Abl-kinase inhibitor than curcumin aimed at the management of colorectal cancer. Consequently, a set of heterocyclic curcumin analogues were designed and docked against the Abl-kinase protein, among which two compounds, i.e., Curcumin and Curcumin semicarbazide indicated superior docking scores. The selected compounds were then additionally estimated for their drug-likeness, solvent accessibility, and free-energy binding, and were found to be within the permissible limits. Auxiliary MD simulation analysis of curcumin semicarbazide revealed the interactions of the ligand with key amino acids as well as a more stable complex when compared to curcumin. These results were further made concrete when tested in vitro in a CRC cell line and semicarbazide curcumin indicated a sevenfold better activity than curcumin. These findings are key to the acceptance of compounds of natural origin to be considered as a small molecule lead in cancer studies. Further in-depth in vitro studies, particularly, gene expression analysis and preclinical animal studies, could inch towards the validation of these findings.

## Supplementary Information

Below is the link to the electronic supplementary material.

Supplementary file1 (PDF 3506 KB)

Supplementary file2 (PDF 3800 KB)
